# How digital health literacy shapes health: Mediating role of physical activity and heterogeneity in China

**DOI:** 10.1371/journal.pone.0328101

**Published:** 2025-07-15

**Authors:** Wei Cheng, Miao Miao, Luying Jiao, Xin Liu

**Affiliations:** 1 School of Education, Beijing Sport University, Beijing, China; 2 Department of Basic Courses, Shanxi Youth Vocational College, Taiyuan, Shanxi, China; Shiraz University of Medical Sciences, IRAN, ISLAMIC REPUBLIC OF

## Abstract

**Purpose:**

This study systematically examines the impact of digital health literacy (DHL) on the physical and mental health of Chinese adults, particularly focusing on how DHL might enhance physical health through physical activity (PA) mediation.

**Methods:**

Drawing on nationally representative Chinese data, this study employed multivariable linear regression combined with mediation analysis to assess the association between DHL and adult health outcomes, while evaluating the mediating role of PA. Robustness was verified through propensity score matching (PSM), logistic regression, and mediation effect pathway tests. Heterogeneity analyses stratified by gender, household registration status, and other demographic factors further elucidated variations in DHL’s impacts on physical and mental health.

**Results:**

DHL demonstrated a positive association with physical health outcomes, while no statistically significant association was observed with mental health. PA served as a partial mediator in the relationship between DHL and physical health enhancement. Stratified analyses revealed significant beneficial effects of DHL on physical health in the following subgroups: males, adults aged ≥60 years, individuals with agricultural household registration, those attaining junior high school education or higher, high-income populations, and participants with health insurance and pension coverage.

**Conclusion:**

This study substantiates the critical influence of DHL on physical health advancement among Chinese adults. Policy formulation should strategically integrate (1) standardized DHL education frameworks, (2) demographically customized digital health management systems, and (3) expanded insurance coverage with optimized reimbursement mechanisms for healthcare and pension schemes. These strategies collectively aim to elevate DHL proficiency, enhance digital platform operational competencies, foster sustainable health-promoting behaviors, and ensure stable health-life equilibrium within China’s adult demographic.

## Introduction

Health is defined as a state of complete physical, mental, and social well-being that extends beyond the mere absence of disease or infirmity [1]. As a core dimension of individual quality of life, health status directly influences the quality of social and economic development. United Nations reports reveal two structural transformations in global population dynamics: First, intensified population aging worldwide, with the proportion of individuals aged 65 + projected to increase from 10% in 2022 to 16% by 2050 [2]; Second, decelerated global population growth driven by declining fertility rates, particularly in European and North American countries where annual population growth has remained below 1% since the mid-1960s [3]. China, as a representative developing nation, recorded a negative natural population growth rate of −0.6% in 2022, with 19.8% of its population aged 60+ [4]. Existing studies confirm that deepening aging and negative population growth may impede economic growth by reducing labor supply quality [5–7]. Given that health status constitutes a critical determinant of work efficiency [8], adult population health exerts substantial impacts on national economic development [9–11].

As an emerging approach to health promotion, DHL refers to the capacity to seek, comprehend, evaluate, and apply health information from digital resources to address health concerns [12]. Empirical investigations have demonstrated significant associations between digital competence and health outcomes [13,14]. The underlying mechanisms may involve health awareness-driven information acquisition behaviors, including accessing psychological knowledge [15,16] and exercise prescriptions [17], which subsequently induce health behavior modifications [18,19] and ultimately enhance self-health management [20]. Building on this evidence, DHL likely influences health status through behavioral engagement mechanisms such as PA participation and dietary improvements [21,22].

The relationship between DHL and physical health has been substantiated by Zangger et al.‘s systematic review, which demonstrated that DHL interventions significantly improved physical functioning metrics [23]. Their follow-up study analyzing 34,000 New Zealand residents using three eHLQ scales further identified a positive correlation between higher DHL levels and better physical health outcomes [24]. In the mental health domain, a randomized controlled trial by Ebert et al. involving a German sample revealed that internet-based stress management interventions significantly reduced symptom severity of depression and anxiety [25]. Similarly, Balay-odao et al.’s study of Saudi nursing students established a significant association between the eHealth Literacy Scale (eHEALS) scores and positive mental health scale (PMHS) ratings [26]. However, conflicting findings from Kim et al. showed no significant link between eHEALS scores and health behaviors in specific populations [27]. These inconsistencies suggest that DHL-health outcome relationships may be moderated by study populations, measurement tools, and contextual factors.

Multidimensional disparities in DHL across demographic groups are well-documented. In terms of gender, Jackson et al. and Wasserman and Richmond-Abbott identified fundamental differences in technology-use motivations and cognitive schemas between males and females, which directly shape their engagement domains and intensity of digital technology utilization [28,29]. Age-related patterns reveal that younger populations exhibit more proactive health information-seeking and integration behaviors, leveraging their superior online information-processing capabilities [30]. In contrast, older adults face dual challenges: the interaction between high chronic disease prevalence and low eHealth literacy not only reduces their digital health service efficacy but also creates negative feedback mechanisms that suppress engagement willingness [31–33]. Spatial disparities manifest in urban-rural DHL gaps rooted in infrastructure inequalities, with cross-national comparisons indicating significantly higher DHL levels in developed versus developing countries [34,35]. Collectively, these systematic findings establish a robust model linking demographic characteristics to DHL variations.

While significant progress has been made in understanding the relationship between DHL and adult health outcomes, three critical methodological and theoretical limitations persist in current research frameworks. First, although multiple studies have independently validated the unidimensional effects of DHL on physical health [36,37] and mental health [38], research paradigms incorporating both health dimensions within a unified measurement system remain underdeveloped. This design limitation may obscure accurate assessment of DHL’s composite effects. Second, extant empirical studies exhibit pronounced geographic sampling bias. While predominant research focuses on urban samples from developed countries, investigations into DHL in resource-constrained regions—particularly rural populations in developing countries—remain conspicuously absent. Addressing this gap could advance precision policy interventions to mitigate health resource disparities among vulnerable groups. Third, insufficient interdisciplinary integration in pathway analysis hampers theoretical advancement. Current research rarely adopts the integrated “knowledge acquisition–behavioral change–welfare enhancement” framework from psychology and behavioral sciences [39,40], constraining innovation in health promotion mechanisms. To address these gaps, this study employs a multi-mediation model to systematically identify transmission pathways linking health cognition adaptation and behavioral modification to DHL-health outcome relationships, thereby contributing novel empirical evidence to refine digital health theory.

## Methods

### Data sources

The study utilized data from the China General Social Survey (CGSS), designed and administered by the National Survey Research Center at Renmin University of China. The CGSS employs stratified, multi-stage sampling with probability proportional to size (PPS), covering all major provinces, autonomous regions, and municipalities in China. To align with the research focus and ensure data currency, the 2021 survey wave was specifically selected. The questionnaire encompasses respondents’ demographic characteristics, physical and mental health status, DHL levels, and PA patterns. After excluding samples with significant missing values, the final analytic sample comprised 1,814 valid observations.

### Ethics statement

This study involved secondary analysis of anonymized public-use data with no direct contact with participants. In accordance with the ethical exemption provisions issued by the Central People’s Government of the People’s Republic of China [41] and the data usage policy of the National Survey Research Center at Renmin University of China [42], ethical approval was not required. However, the study also obtained ethical approval from the Ethics Committee of Shanxi Youth Vocational College.

### Core independent variables

The independent variable is Chinese adults’ DHL. Based on the CGSS instrument, seven items were selected, including “Do you often search for information about healthy lifestyle on the internet?” Each item was measured on a 5-point Likert scale (1 = Never, 5 = Very frequently). These secondary indicators were synthesized into a primary DHL index using principal component analysis (PCA), with higher scores indicating greater DHL. The full list of items is presented in Table 1.

**Table 1:** Measurement indicators for DHL.

Primary indicator	Secondary indicators
**DHL**	Do you often search for information about healthy lifestyle on the internet?
	Do you often search for information about vaccine immunization on the internet?
	Has the information on the internet had a positive impact on my health behaviors in the past 12 months?
	Has the information on the internet helped me understand what my doctor has told me in the past 12 months?
	Can the internet help people determine if they need to see a doctor?
	Does the internet help confirm whether doctors have given people appropriate advice?
	Is it difficult to distinguish the reliability of health information on the internet?

### Dependent variable

The dependent variable is Chinese adults’ physical and mental health status. For physical health, responses to the item “How would you rate your current physical health status?” were assessed on a 5-point scale (1 = Very unhealthy, 5 = Very healthy), where higher values denote better physical health. Mental health was evaluated using the item “How frequently have you felt depressed or downhearted in the past four weeks?” scored inversely on a 5-point scale (1 = Always, 5 = Never), with higher scores reflecting better mental health outcomes.

### Mediating variables

The mediating variable in this study was the frequency of physical exercise among Chinese adults. Participants were assessed using the question: “During the past year, have you regularly engaged in physical exercise during leisure time?” Responses were recorded on a 5-point scale (1 = Never, 5 = Daily), with higher scores indicating greater exercise frequency. A second question—”Do you regularly engage in physical exercise lasting at least 20 minutes that causes sweating or accelerated breathing?”—was employed for robustness checks, using the same 5-point scale and scoring interpretation.

### Control variables

Following established methodological approaches for covariate selection (Baron & Kenny, 1986), we included the following control variables: gender (1 = female, 0 = male); age (calculated by subtracting birth year from survey year); agricultural household registration status (1 = yes, 0 = no); marital status (1 = married, 0 = unmarried); employment status (1 = employed, 0 = unemployed); health insurance coverage (1 = insured, 0 = uninsured); pension insurance coverage (1 = enrolled, 0 = not enrolled); annual income; and years of formal education.

### Model specification and analysis procedures

To estimate the impact of DHL on health outcomes among adults, this study employs a multiple regression model as follows:


$$Y_{i}=\alpha_{0}+\alpha_{1}{DHL}_{i}+\sum_{\beta }X_{i}+\varepsilon_{i}$$

Here, $Y_{i}$ represents the health level of adults. ${DHL}_{i}$denotes individual DHL, while $X_{i}$ encompasses a set of control variables, including gender, age, household registration status, marital status, years of education, annual income, employment status, and the availability of medical and pension insurance. $\varepsilon_{i}$ denotes the individual-level random error term. The coefficient $\alpha_{1}$ captures the association between DHL and health outcomes.

### Testing for moderated mediation

The moderated mediation analysis follows the approach proposed by Baron and Kenny [43], with the following models:


$${PH}_{i}=\alpha_{0}+\alpha_{1}{DHL}_{i}+\alpha_{2}X_{i}+\varepsilon_{1}$$ (1)

$${PA}_{i}=\beta_{0}+\beta_{1}{DHL}_{i}+\beta_{2}X_{i}+\varepsilon_{2}$$ (2)

$${PH}_{i}=\gamma_{0}+\gamma_{1}{DHL}_{i}+\gamma_{2}{PA}_{i}+\gamma_{3}X_{i}+\varepsilon_{3}$$ (3)

In these equations, ${PH}_{i}$ represents physical health level, ${DHL}_{i}$ denotes DHL, and ${PA}_{i}$ signifies PA. $X_{i}$ refers to the full set of control variables, while *α、β、γ* represent regression coefficients. $\varepsilon_{i}$ denotes the random disturbance term. Equation (1) examines the direct effect of DHL on physical health. Equation (2) assesses the relationship between DHL and PA. Equation (3) tests whether PA mediates the association between DHL and physical health, with potential moderation effects considered.

## Results

Table 2 presents the key demographic characteristics of Chinese adults in the sample. The standardized mean scores for physical health and mental health were 3.65 (SD = 0.99) and 4.02 (SD = 1.02), respectively. Participants had a mean age of 45.5 years with nearly balanced gender distribution (male 45.2%, female 54.8%). Rural residents accounted for 65.6% of the sample, while 69.6% were married. The average educational attainment reached 10.8 years, with 54.3% currently employed and a mean annual income of ¥49,425.8. Insurance coverage rates stood at 94.8% for medical insurance and 69% for pension insurance.

**Table 2:** Description of basic characteristics of adults (N = 1814).

	Mean	SD	Median	Min	Max
**Dependent variables**					
**Physical health**	3.657	0.99	4	1	5
**Mental health**	4.027	1.026	4	1	5
**Independent variables**					
**DHL**	0.003	1.581	0.153	−5.678	4.759
**Intermediate variables**					
**PA 1**	3.007	1.537	3	1	5
**PA 2**	3.164	1.68	3	1	5
**Control variables**					
**Gender**	0.452	0.498	0	0	1
**Age**	45.525	16.574	45	18	88
**Rural household registration**	0.656	0.475	1	0	1
**Marital Status**	0.696	0.46	1	0	1
**Education**	10.803	4.274	12	0	19
**Income**	49425.849	71584.79	30000	0	600000
**Employed**	0.543	0.498	1	0	1
**Medical Insurance**	0.948	0.223	1	0	1
**Endowment Insurance**	0.69	0.463	1	0	1

Descriptive statistics revealed that Chinese adults generally exhibited favorable physical and mental health status, while demonstrating substantial individual variations in DHL. Specifically, DHL showed a mean score of 0.003 (SD = 1.581), indicating significant dispersion across the population. PA engagement appeared relatively infrequent, with two key indicators revealing limited exercise participation: The item measuring “Frequency of leisure-time physical exercise” (PA 1) yielded a mean score of 3.01, while the assessment of “Regular engagement in ≥20-minute vigorous exercise inducing perspiration or accelerated breathing” (PA 2) showed a mean of 3.16. These quantitative findings suggest that most adults participated in PA only a few times monthly.

### Regression analysis

This study employed multiple linear regression (OLS) models to examine DHL’s impact on adult health outcomes, with detailed results presented in Table 3. The baseline specifications (Columns 1 and 3) revealed a statistically significant positive association between DHL and physical health (β = 0.085, p < 0.01), while no significant relationship emerged with mental health (β = −0.010, p > 0.1). After incorporating control variables including gender, age, household registration type, marital status, education, and income (Columns 2 and 4), DHL maintained its significant association with physical health (β = 0.038, p < 0.05). Notably, the standardized coefficients indicate that a one-standard-deviation increase in DHL corresponded to a 0.038-point elevation in physical health scores, equivalent to 3.8% of the total standard deviation.

**Table 3:** The impact of DHL on physical and mental health of adults.

Variables	Physical health	Physical health	Mental health	Mental health
**DHL**	0.085^***^	0.038^**^	−0.01	−0.025
	(−0.015)	(−0.016)	(−0.015)	(−0.017)
**Gender**		0.056		0.173^***^
		(−0.048)		(−0.053)
**Age**		−0.016^***^		−0.001
		(−0.002)		(−0.002)
**Agricultural household registration**		0.065		−0.054
		(−0.05)		(−0.056)
**Marital Status**		0.092		0.218^***^
		(−0.059)		(−0.064)
**Education**		0.036^***^		0.033^***^
		(−0.007)		(−0.007)
**Income**		0		0.000^***^
		0		0
**Employed**		0.073		−0.061
		(−0.053)		(−0.057)
**Medical Insurance**		−0.107		0.06
		(−0.105)		(−0.123)
**Endowment Insurance**		0.032		0.064
		(−0.057)		(−0.063)
**Constant**	3.657^***^	3.906^***^	4.028^***^	3.448^***^
	(−0.023)	(−0.167)	(−0.024)	(−0.191)
**Observations**	1,814	1,611	1,811	1,609
**R-squared**	0.018	0.141	0	0.038

*** p < 0.01,

** p < 0.05,

* p < 0.1, Standard errors in parentheses.

Among control variables, educational attainment demonstrated significant positive associations with both physical (β = 0.036, p < 0.01) and mental health (β = 0.033, p < 0.01). Age exhibited a negative relationship with physical health (β = −0.016, p < 0.01). Gender (β = 0.173, p < 0.01) and marital status (β = 0.218, p < 0.01) showed significant mental health associations but no physical health linkages. Model diagnostics indicated that covariate inclusion substantially improved the explanatory power for physical health outcomes, with R² values increasing from 0.018 to 0.141.

### Mediation effect analysis

To examine the mediating role of PA in the relationship between DHL and physical health, this study implemented a three-step regression framework (Table 4). All models adjusted for covariates (e.g., gender, age) and utilized robust standard errors. Direct effect analysis (Model 1): DHL demonstrated a statistically significant positive association with physical health (β = 0.038, p < 0.05). Mediation pathway analysis (Model 2): Higher DHL significantly predicted increased PA frequency (β = 0.117, p < 0.01). Integrated mediation test (Model 3): Inclusion of PA attenuated the DHL coefficient from 0.038 to 0.033 (p < 0.05), while PA itself exhibited a significant positive association with physical health (β = 0.046, p < 0.01). Model explanatory power improved marginally (ΔR² = 0.005, from 0.141 to 0.146). Sobel mediation testing revealed a partial mediation effect, with PA accounting for 14.16% of the total association (indirect effect = 0.0043).

**Table 4:** The mediating effect of PA on the relationship between DHL and physical health.

Variables	Physical health	PA	Physical health
**DHL**	0.038^**^	0.117^***^	0.033^**^
	(−0.015)	(−0.023)	(−0.015)
**Exercise frequency**			0.046^***^
			(−0.016)
**Constant**	3.906^***^	1.902^***^	3.826^***^
	(−0.165)	(−0.264)	(−0.168)
**Observations**	1,611	1,610	1,609
**R-squared**	0.141	0.114	0.146
**Control variables**	Yes	Yes	Yes

*** p < 0.01, ^**^ p < 0.05, ^*^ p < 0.1 Standard errors in parentheses

### Heterogeneity analysis

Stratified regression analyses assessed subgroup variations in DHL’s association with physical health, incorporating controls for gender, age, household registration type, marital status, education, income, employment status, and health/pension insurance coverage (Table 5). The results demonstrated significant positive associations among males (β = 0.047, p < 0.1), adults aged ≥60 years (β = 0.036, p < 0.1), agricultural household registrants (β = 0.043, p < 0.05), individuals with junior high education or higher (β = 0.056, p < 0.01), high-income earners (β = 0.041, p < 0.05), and insurance beneficiaries (health insurance: β = 0.044, p < 0.01; pension insurance: β = 0.058, p < 0.01). In contrast, no statistically significant relationships were observed for female participants, non-agricultural registrants, those with below-junior-high education, low-income individuals, or uninsured populations. The model explanatory power ranged from R² = 0.083 to 0.221 across subgroups, indicating substantial variation in health impact mechanisms among different population strata.

**Table 5:** Heterogeneity analysis examining the impact of DHL on physical health.

		DHL		Control variables		Constant	Observations	R²
**Male**	0.047^*^		(−0.024)	YES	4.054^***^	(−0.269)	737	0.131
**Female**	0.03		(−0.022)	YES	3.895^***^	(−0.225)	874	0.155
**over 60 years old**	0.031		(−0.032)	YES	2.536^***^	(−0.647)	353	0.113
**under 60 years old**	0.036^*^		(−0.018)	YES	3.973^***^	(−0.196)	1258	0.095
**Agricultural household registration**	0.043^**^		(−0.021)	YES	3.931^***^	(−0.201)	1046	0.166
**Non-agricultural household registration**	0.022		(−0.025)	YES	4.129^***^	(−0.252)	565	0.117
**Middle school and above**	0.056^***^		(−0.022)	YES	4.009^***^	(−0.315)	811	0.083
**Below Middle school**	0.016		(−0.023)	YES	3.930^***^	(−0.243)	800	0.148
**Income above the median (¥20,000 and above)**	0.041^**^		(−0.018)	YES	3.889^***^	(−0.22)	1029	0.095
**Income below the median (under ¥20,000)**	0.019		(−0.03)	YES	4.028^***^	(−0.299)	582	0.187
**With medical insurance**	0.044^***^		(−0.017)	YES	3.766^***^	(−0.153)	1524	0.148
**Without medical insurance**	−0.006		(−0.059)	YES	4.412^***^	(−0.601)	87	0.145
**With endowment insurance**	0.058^***^		(−0.019)	YES	3.750^***^	(−0.242)	1148	0.129
**Without endowment insurance**	−0.019		(−0.028)	YES	4.514^***^	(−0.274)	463	0.221

*** p < 0.01, ^**^ p < 0.05, ^*^ p < 0.1 Standard errors in parentheses

Population heterogeneity in the association between DHL and mental health was systematically examined across demographic subgroups following full covariate adjustment, with analytical outcomes detailed in Table 6. The analysis revealed significant negative associations among males (β = −0.069, p < 0.01), adults aged below 60 years (β = −0.048, p < 0.05), high-income individuals (β = −0.043, p < 0.05), and populations without pension insurance coverage (β = −0.085, p < 0.01). In contrast, no statistically meaningful relationships were observed for female participants, adults aged ≥60 years, agricultural/non-agricultural household registrants, individuals across educational attainment levels, or those with/without health insurance. The models demonstrated varying explanatory power (R² = 0.018–0.13), suggesting distinct mechanistic pathways underlying mental health outcomes that warrant further investigation.

**Table 6:** Heterogeneity analysis examining the impact of DHL on mental health.

	DHL		Control variables	Constant		Observations	R²
**Male**	−0.069***	(−0.024)	YES	3.728***	(−0.279)	736	0.026
**Female**	0.006	(−0.024)	YES	3.321***	(−0.283)	873	0.049
**over 60 years old**	0.025	(−0.034)	YES	2.853***	(−0.772)	353	0.13
**under 60 years old**	−0.048**	(−0.02)	YES	3.691***	(−0.222)	1256	0.026
**Agricultural household registration**	−0.019	(−0.021)	YES	3.297***	(−0.214)	1045	0.054
**Non-agricultural household registration**	−0.045	(−0.028)	YES	3.888***	(−0.344)	564	0.038
**Junior high school and above**	−0.028	(−0.024)	YES	3.904***	(−0.343)	810	0.018
**Below junior high school**	−0.032	(−0.024)	YES	3.312***	(−0.29)	799	0.071
**Income above the median (¥20,000 and above)**	−0.043**	(−0.02)	YES	3.711***	(−0.241)	1028	0.033
**Income below the median (under ¥20,000)**	−0.008	(−0.03)	YES	3.468***	(−0.34)	581	0.054
**With medical insurance**	−0.022	(−0.018)	YES	3.462***	(−0.157)	1522	0.041
**Without medical insurance**	−0.048	(−0.062)	YES	4.392***	(−0.793)	87	0.082
**With endowment insurance**	−0.005	(−0.02)	YES	3.362***	(−0.267)	1146	0.04
**Without endowment insurance**	−0.085***	(−0.031)	YES	3.508***	(−0.345)	463	0.06

*** p < 0.01, ** p < 0.05, * p < 0.1, Robust standard errors in parentheses.

### Robustness check

The positive impact of DHL on physical health and the robustness of physical exercise’s mediating effects were systematically validated through a tripartite analytical approach: propensity score matching, logit regression modeling, and mediation pathway verification.

Robustness Check Method 1: Propensity Score Matching. The effects of DHL on physical and mental health were validated through three approaches—nearest neighbor matching (NNM), radius matching (RM), and kernel density matching (KDM)—with results consistent with the primary regression findings. This indicates that DHL significantly enhances physical health (coefficients ranging from 0.012 to 0.025, all significant at the 1% level), while its impact on mental health remains negative and statistically insignificant. Detailed outcomes are presented in Table 7.

**Table 7:** The impact of DHL on physical and mental health using propensity value matching method.

Variables	NNM	NNM	RM	RM	KDM	KDM
Physical health	Mental health	Physical health	Mental health	Physical health	Mental health
**DHL**	0.025***	−0.096	0.019***	−0.108	0.012***	−0.112
	(−0.011)	(−0.062)	(−0.007)	(−0.059)	(−0.005)	(−0.08)
**Control variables**	YES	YES	YES	YES	YES	YES
**Constant**	3.945***	3.573***	3.993***	3.320***	4.061***	3.419***
	(−0.276)	(−0.293)	(−0.211)	(−0.262)	(−0.207)	(−0.258)
**Observations**	1527	1536	7212	7188	7217	7193
**R-squared**	0.197	0.108	0.174	0.089	0.163	0.079

*** p < 0.01, ** p < 0.05, * p < 0.1, Robust standard errors in parentheses.

Robustness Check Method 2: Logit Regression Analysis. The influence of DHL on physical and mental health was re-examined using logit regression, with results summarized in Table 8. By transforming the primary variables into binary categorical measures, the analysis reaffirmed the earlier findings: DHL exerts a statistically significant positive effect on physical health, with coefficients of 0.139^***^ and 0.065^*^ for physical health outcomes, significant at the 1% and 5% levels, respectively.

**Table 8:** Effects of DHL on physical and mental health using logit regression.

Variables	Physical health	Physical health	Mental health	Mental health
	(Binary variable)	(Binary variable)	(Binary variable)	(Binary variable)
**DHL**	0.139***	0.065*	0.029	−0.027
	(−0.031)	(−0.034)	(−0.036)	(−0.04)
**Control variables**	YES	YES	YES	YES
**Constant**	0.381***	1.011***	0.319***	0.086
	(−0.048)	(−0.389)	(−0.057)	(−0.441)
**Observations**	1814	1611	1277	1132

*** p < 0.01, ** p < 0.05, * p < 0.1,Robust standard errors in parentheses.

Robustness Check Method 3: Mediation Analysis of PA. To further validate the mediating role of PA in the relationship between DHL and physical health, a revised measure from the CGSS survey was adopted: “Do you regularly engage in at least 20 minutes of physical exercise that induces sweating or accelerated breathing?” As shown in Table 9, DHL continues to positively predict physical health (coefficient: 0.033, p < 0.05), with PA mediating approximately 11.53% of this effect. The full results are provided in Table 9.

**Table 9:** Test of mediating effects.

Variables	PA (exercise for over 20 minutes)	Physical health
**DHL**	0.086***	0.033**
	(−0.027)	(−0.015)
**Exercise over 20 min**		0.050***
		(−0.014)
**Control variables**	YES	YES
**Constant**	1.925***	3.806***
	(−0.297)	(−0.167)
**Observations**	1,604	1,603
**R-squared**	0.054	0.147

*** p < 0.01, ** p < 0.05, * p < 0.1, Standard errors in parentheses.

## Discussions

This study demonstrates that DHL exhibits a significant positive association with physical health outcomes among Chinese adults, though its direct effects on mental health did not reach statistical significance. Subsequent mediation analyses identified PA as serving a partial mediating role in the DHL-physical health relationship, revealing that the translation of health behaviors constitutes a critical pathway through which digital knowledge acquisition empowers health outcomes.

### DHL’s impact on physical health

The observed positive association between DHL and physical health in the Chinese context aligns with findings from systematic reviews conducted in high-income countries [23]. This relationship receives further cross-cultural support from Zangger et al.‘s empirical study in New Zealand, which clarifies the universal mechanism by which DHL enhances physical health through health behavior optimization, particularly via increased PA [24]. Furthermore, analysis of longitudinal panel data from three waves (2016, 2018, and 2020) from the China Family Panel Study confirmed that digital literacy significantly improves physical health outcomes [44]. Collectively, this consistency across international and domestic studies highlights the critical role of health information empowerment strategies in advancing population health.

### Mediating role of PA

The identified mediating pathway through PA in the DHL-physical health relationship substantiates the “knowledge acquisition—behavioral modification—well-being” framework. This conceptual framework integrates principles from cognitive-behavioral and exercise science theories, with the “knowledge-behavior” transition aligning with cognitive behavioral therapy (CBT) postulates regarding the interdependence of cognition, affect, and behavior, where cognitive restructuring enables targeted behavioral adaptations [45,46]. Within this construct, enhanced DHL competencies facilitate systematic health knowledge integration in adults, driving cognition-driven behavioral strategies for health management and problem prevention [21]. A cross-national study conducted in Germany and Austria supports this pathway by demonstrating that individuals with higher eHealth literacy are more likely to develop sustained healthy habits, such as engaging in regular PA, when accompanied by strong self-efficacy, that serves as a cognitive enabler for behavior change in digital health contexts [47].

The “behavior-wellness” linkage corresponds with evidence on health outcome optimization through scientifically structured physical exercise [40]. As a measurable manifestation of health-promoting behaviors [22], sustained engagement in evidence-based PA demonstrates longitudinal benefits for maintaining functional health status [39]. This relationship is further supported by large-scale studies from Western populations, which confirm that leisure-time PA yields significant health gains, including lower all-cause mortality and improved quality of life. A pooled analysis of over 660,000 adults in the United States and Europe identified optimal longevity benefits at three to five times the recommended activity levels, with no harm at even higher volumes [48]. Data from a nationwide US survey further showed that adults meeting recommended PA thresholds reported fewer unhealthy days, regardless of physical limitations [49].

These findings provide both theoretical and empirical grounding for developing integrated strategies that pair digital literacy enhancement with tailored PA interventions. To assess the plausibility of alternative behavioral mediators, we conducted an exploratory mediation analysis using two additional variables from the CGSS: binge drinking frequency and fruit and vegetable consumption. While these pathways showed limited effects (with indirect effects of –2.37% and 7.76%, respectively), they reinforce the unique strength and relevance of PA as a behavioral mechanism through which DHL promotes health (see S1 Tables and S2).

### The impact of DHL on mental health

The non-significant association between DHL and mental health may stem from multi-dimensional factors. First, measurement limitations may arise from the use of self-rated indicators such as “frequency of feeling depressed,” which may inadequately capture culturally specific manifestations of psychological distress in Chinese populations, including somatic symptom reporting [50,51]. Supporting this finding, A comparative clinical study revealed that Han Chinese participants reported somatic symptoms during depressive episodes more frequently than Euro-Canadians, reflecting cultural norms in distress expression [52].

Second, sociocultural stigma introduces reporting bias. Psychological disorders remain heavily stigmatized in Chinese society [53], potentially leading to underreporting of symptoms like depression and anxiety. This is exemplified by a community-based study in central China, in which perceived societal stigma toward depression discouraged symptom disclosure, particularly among older adults and males [54].

Third, fragmented digital health infrastructure limits impact. In China, digital mental health services remain underdeveloped in primary and community care settings, with rural populations disproportionately affected [55–57]. Compounding this limitation, a national survey found that awareness and usage of digital platforms for psychological support remain low, especially among older adults and low-income groups [58]. This service gap, combined with insufficient DHL, likely undermines digital tools’ mental health benefits. To address these challenges, future interventions should integrate DHL enhancement with infrastructure expansion and public trust-building—essential steps for implementing effective digital mental health solutions across diverse communities.

### Heterogeneity analysis

Heterogeneity analyses revealed population-specific variations in DHL’s impact on physical health, demonstrating systematic associations with sociodemographic determinants. Gender-specific patterns emerged, with males deriving greater benefits—a finding consistent with Zangger et al.’s observation of male preference for rapid-access digital health platforms [24]. Age-stratified effects showed attenuated benefits among adults ≥60 years, contrasting with younger populations’ enhanced gains attributable to technological proficiency [59] and preventive health orientation [60]. This is supported by studies indicating that younger adults in China demonstrate more autonomous use of health information apps and exhibit greater digital adaptability [61].

Household registration disparities highlighted dual barriers in traditional and digital health access for agricultural household populations [62]. However, selective technological penetration enabled prioritized benefits for educated rural subgroups—suggesting that digital interventions must be tailored to both geographical and cultural contexts [63,64]. Educational attainment and income further moderated effects: individuals with higher education possess more advanced health information appraisal skills, facilitating more effective use of digital content [65] while higher-income groups are more capable of health-related digital consumption [66]. Moreover, medical insurance and endowment insurance coverage were found to enhance individuals’ motivation to engage with digital health services, by lowering their perceived financial risk and access burden [35,67].

### Strengths and limitations

This investigation advances DHL research through three theoretical contributions. Theoretically, it quantifies DHL’s direct effects on physical health via multivariate regression while empirically validating PA’s mediating role—thereby extending e-health literacy models and substantiating the “knowledge acquisition—behavioral modification—well-being” transmission paradigm. Methodologically, stratified regression techniques systematically reveal urban-rural disparities in education’s moderating effects on DHL, providing novel evidence on the digital determinants of health disparities. Culturally, based on the findings of a non-significant association between DHL and mental health in the Chinese context, the constructed cultural sensitivity framework emphasizes the need to re-examine some moderating factors. These factors include the validity of assessment tools, the social impact of stigmatization, and service accessibility. This has enlightenment value for the development of localized digital health intervention strategies.

There are four limitations in the research that call for further improvement. Regarding data, the cross-sectional design cannot capture the delayed effects of digital literacy and the cumulative impacts of health – related behaviors. Even though we’ve done robustness checks using the Bootstrap method, causal inference still needs tracking data for support. This limitation constrains the ability to establish temporal ordering and thus weakens the identification of causal relationships between DHL and health outcomes. Future studies are encouraged to adopt longitudinal or experimental designs to better clarify the temporal sequencing and causal mechanisms underlying the relationship between DHL and health outcomes.

Methodologically, the linear regression model has verified the mediating effect, but it hasn’t analyzed the multi – stage mediating processes. For example, digital literacy influences health information screening, which then leads to behavior change and finally affects health outcomes. Moreover, machine-learning methods have not been used to identify non – linear relationships.To address these limitations, future studies are encouraged to apply multi-stage mediation models and machine learning techniques, which can more accurately reflect the layered and potentially nonlinear relationships between DHL and health outcomes.

In measurement, self-rated indicators for physical and mental health, as well as for PA and DHL, are susceptible to recall bias, cultural cognitive biases, and social desirability effects. For instance, the elderly might overestimate their physical health, and due to the stigma around mental health, the reporting rate of depressive symptoms is low. Additionally, the CGSS 2021 dataset lacks objective indicators such as data from wearable devices to provide more precise assessments of PA. While our analysis included all available DHL-related items, the use of only seven survey questions may be insufficient to fully capture the complexity and evolving nature of DHL, especially considering rapid technological advancements such as Web 3.0. Future research should employ multidimensional and validated instruments for DHL to better address these measurement limitations.

With regard to the explanation of mechanisms, we additionally examined two additional behavioral mediators—binge drinking and the intake of fresh fruits and vegetables—based on available items from the CGSS 2021 dataset, to test the robustness of PA as a key mediating pathway between DHL and physical health outcomes. The mediation effects of these two additional variables were relatively limited, with shares of –2.37% and 7.76%, respectively, compared to 14.16% for PA. While this finding reinforces the central role of PA, it does not imply that the underlying mechanisms have been exhaustively explored. For instance, due to missing values in the CGSS 2021 dataset, potentially important moderators such as social support networks could not be incorporated. The absence of these factors may limit a more comprehensive interpretation of how digital interventions influence adult health, especially through socially embedded mechanisms.

## Conclusion

This study provides empirical evidence on how DHL contributes to physical health among Chinese adults, partly through increased PA. While DHL showed no significant direct effect on mental health, the findings suggest that physical health benefits are more pronounced among males, older adults, individuals with agricultural household registration, those with higher education levels, and those covered by insurance. These results underscore the need for targeted DHL interventions that address demographic disparities. Given the limitations of a cross-sectional design and self-reported measures, future longitudinal and mixed-methods research is warranted to clarify causal mechanisms and inform precision public health strategies in digital contexts.

## Supporting information

S1 Table Regression Results of DHL, Drinking, and Physical Health. (DOCX)

S2 Table Regression Results of DHL, Fruit and Vegetable Intake, and Physical Health. (DOCX)
