# Supplementary material for: How digital health literacy shapes health: Mediating role of physical activity and heterogeneity in China
Source: PLoS One. 2025 Jul 15;20(7):e0328101. doi: 10.1371/journal.pone.0328101 (PMC12262867; doi:10.1371/journal.pone.0328101)
Supplement: S2 Table — (DOCX) [file pone.0328101.s002.docx]

| **Table S2. Regression Results of DHL, Fruit and Vegetable Intake, and Physical Health** | | | |
| --- | --- | --- | --- |
| **Variables** | **Physical health(1)** | **Fruit and Vegetable Intake(2)** | **Physical Health(3)** |
| **Drinking** |  |  | 0.159*** |
|  |  |  | (0.030) |
| **DHL** | 0.037** | 0.018 | 0.034** |
|  | (0.015) | (0.012) | (0.015) |
| **Gender** | 0.047 | -0.156*** | 0.072 |
|  | (0.048) | (0.040) | (0.048) |
| **Age** | -0.016*** | 0.003* | -0.016*** |
|  | (0.002) | (0.002) | (0.002) |
| **Agricultural Household Registration** | 0.072 | 0.002 | 0.072 |
|  | (0.052) | (0.043) | (0.051) |
| **Marital Status** | 0.086 | 0.147*** | 0.063 |
|  | (0.055) | (0.046) | (0.054) |
| **Education** | 0.033*** | 0.025*** | 0.029*** |
|  | (0.007) | (0.005) | (0.007) |
| **Income** | 0.000** | -0.000 | 0.000** |
|  | (0.000) | (0.000) | (0.000) |
| **Employed** | 0.054 | -0.037 | 0.059 |
|  | (0.053) | (0.044) | (0.052) |
| **Medical Insurance** | -0.097 | -0.147* | -0.073 |
|  | (0.104) | (0.086) | (0.103) |
| **Endowment Insurance** | 0.023 | 0.058 | 0.013 |
|  | (0.056) | (0.047) | (0.056) |
| **Constant** | 3.912*** | 3.267*** | 3.393*** |
|  | (0.165) | (0.138) | (0.191) |
| **Observations** | 1,611 | 1,611 | 1,611 |
| **R-squared** | 0.143 | 0.036 | 0.158 |
| *** p<0.01, ** p<0.05, * p<0.1, Standard errors in parentheses | | | |
